# Pulse granuloma: a rare condition mimicking a gastric tumor

**DOI:** 10.1590/0100-3984.2015.0058

**Published:** 2016

**Authors:** Maurício Fabro, Sara Raquel Fabro, Rafael Santiago Oliveira de Sales, Luiz Pedro de Souza Júnior, Julian Catalan

**Affiliations:** 1 Hospital Santa Catarina de Blumenau, Blumenau, SC, Brazil.

*Dear Editor*,

We report the case of a 60-year-old female patient who reported a one-week history of pain in the left hypochondrium, fever, vomiting, and diarrhea. The physical examination and laboratory tests showed no significant changes. Ultrasound showed a septated cystic mass, alongside the stomach, with thick walls and containing debris, although without any vascularity seen on the Doppler flow study (Figure 1A). For clarification, we performed computed tomography (CT), which identified an expansive parietal lesion in the gastric body, measuring 5.9 × 4.5 cm, with contrast uptake by the walls and septa, especially in the portal phase, together with a hypointense central component without enhancement, suggestive of necrosis (Figure 1B). The diagnostic hypotheses were gastric adenocarcinoma and gastrointestinal stromal tumor. The patient underwent upper gastrointestinal endoscopy, which showed an elevated lesion in the greater curvature of the stomach, with irregular, ulcerated mucosa (Figure 2A). A biopsy yielded inconclusive results, and we opted for resection of the lesion. Histopathological examination of the specimen demonstrated pulse granuloma (Figure 2B). The patient was discharged on the fifth postoperative day, with subsequent outpatient follow-up.

**Figure 1:**
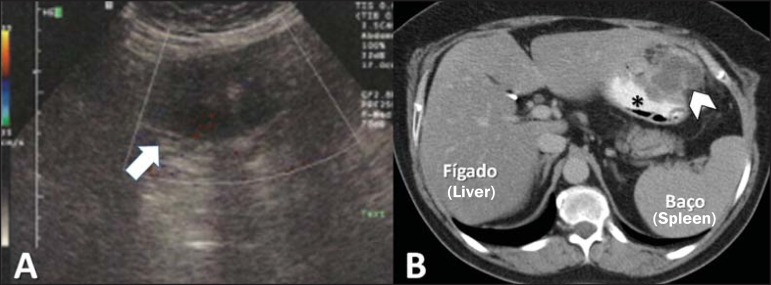
**A:** Ultrasound showing a septated cystic mass in the left hypochondrium without vascularity on the Doppler flow study (arrow). **B:** CT of the abdomen, showing a mass in the stomach wall (arrowhead). Stomach filled with contrast material (asterisk).

**Figure 2:**
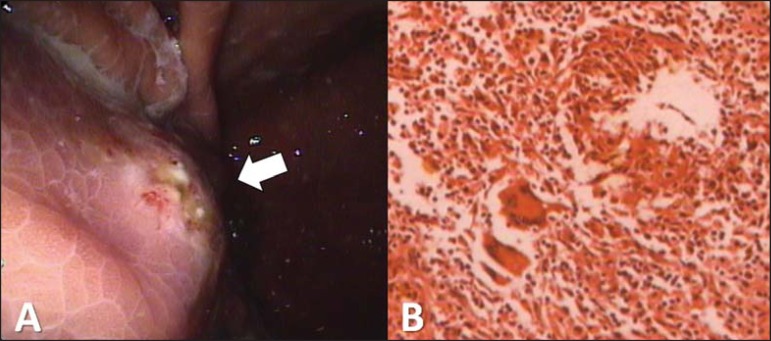
**A:** Upper gastrointestinal endoscopy showing an expansive lesion with irregular, ulcerated mucosa in the greater curvature of the gastric body (arrow). **B:** Photomicrograph showing a granulomatous inflammatory process, at some points arranged in a palisade, with numerous multinucleated foreign-body giant cells.

Pulse granuloma is a benign lesion^(1)^ that is extremely rare^(2,3)^. It was first described in 1969 by Knoblich, who characterized it as lung injury^(4)^. Lewars described the first oral lesion in 1971^(5)^, the initial reports of the disease in the extraoral gastrointestinal tract not appearing until 2001^(6)^.

Pulse granuloma is characterized by a chronic granulomatous reaction to a foreign body of vegetable origin^(1)^, typically indigestible cellulose deposited under the mucosa^(3)^. Most pulse granuloma patients have a history of bowel disease, including diverticulitis, fistula, perforation, ulcerative colitis, appendicitis, or anastomotic leakage^(7)^, allowing the foreign body to reach the deep layers of the intestinal wall. The oral cavity is the site most often affected, the occurrence of pulse granuloma at other sites being extremely rare^(3)^. However, there have been reports of pulse granuloma in the stomach, small intestine, colon, peritoneum, mesentery, genitourinary tract, and skin^(2,7)^.

Pulse granuloma predominantly affects males^(7)^, of a broad range of ages, cases having been described in patients from 13 to 85 years of age^(7)^. The symptoms are vague and nonspecific^(8)^, occasionally including abdominal pain and discomfort^(2)^.The physical examination is usually unremarkable, although a palpable mass can be identified^(2,7)^.

The imaging evaluation of pulse granuloma is usually made either by ultrasound, the findings of which are often nonspecific, or by CT, which is more relevant because of its high sensitivity and specificity for the detection and characterization of foreign bodies in the gastrointestinal tract^(8)^. Nevertheless, because foreign bodies of vegetable origin do not produce hyperintense images, the diagnosis is not usually obtained by CT. Upper gastrointestinal endoscopy is a useful tool in the study of gastric lesions and allows the collection of material for histopathological evaluation. However, endoscopic biopsies are usually small and superficial, which can make it difficult to confirm the diagnosis of granuloma pulse^(8)^. The diagnosis is made through exclusion on the basis of the histopathological findings^(1)^. The possibility of pulse granuloma should be considered in cases of expansive lesions in the gastrointestinal tract^(8)^, the main differential diagnoses being adenocarcinoma, gastrointestinal stromal tumor, and leiomyoma^(8)^. The definitive treatment is surgical intervention^(1)^.

## References

[1] Razavi A, Vlcek D, Kutten-Berger JJ (2014). Oral pulse granuloma of the mandible - a case
report. Swiss Dent J.

[2] Geramizadeh B, Mousavi SJ, Bananzadeh A (2014). Omental mass caused by pericolic vegetable granuloma: a rare case
report. Ann Colorectal Res.

[3] Yeo NK, Eom DW, Lim HW (2014). Vegetable or pulse granuloma in the nasal cavity. Clin Exp Otorhinolaryngol.

[4] Knoblich R (1969). Pulmonary granulomatosis caused by vegetable particles. So-called
lentil pulse pneumonia. Am Rev Respir Dis.

[5] Lewars PH (1971). Chronic periostitis in the mandible underneath artificial
dentures. Br J Oral Surg.

[6] Rhee DD, Wu ML (2006). Pulse granulomas detected in gallbladder, fallopian tube, and
skin. Arch Pathol Lab Med.

[7] Nowacki NB, Arnold MA, Frankel WL (2015). Gastrointestinal tract-derived pulse granulomata: clues to an
underrecognized pseudotumor. Am J Surg Pathol.

[8] Shan GD, Chen ZP, Xu YS (2014). Gastric foreign bogy granuloma caused by an embedded fishbone: a
case report. World J Gastroenterol.

